# Urbanicity and psychotic disorders: Facts and hypotheses

**DOI:** 10.1080/19585969.2023.2272824

**Published:** 2023-11-23

**Authors:** Baptiste Pignon, Andrei Szöke, Benson Ku, Maria Melchior, Franck Schürhoff

**Affiliations:** a AP-HP, Hôpitaux Universitaires “H. Mondor”, DMU IMPACT, INSERM, IMRB, translational Neuropsychiatry, Fondation FondaMental, Univ Paris-Est-Créteil (UPEC), Créteil, France; b Department of Psychiatry and Behavioral Sciences, Emory University School of Medicine, Atlanta, GA, USA; c Sorbonne Université, INSERM, Institut Pierre Louis d‘Épidémiologie Et de Santé Publique, IPLESP, Equipe de Recherche en Epidémiologie Sociale, ERES, Paris, France

**Keywords:** Urbanicity, psychotic disorders, schizophrenia, psychosis, psychotic symptoms, air pollution, psychosocial stress

## Abstract

In the present qualitative literature review, we summarise data on psychotic disorders and urbanicity, focusing particularly on recent findings. Longitudinal studies of the impact of urbanicity on the risk for psychotic disorders have consistently shown a significant association, with a relative risk between 2 and 2.5. However, most of the original studies were conducted in Western Europe, and no incidence studies were conducted in low- and middle-income countries. European studies suggest that neighbourhood-level social fragmentation and social capital may partly explain this association. Exposure to air pollution (positive association) and green space (negative association) may also be part of the explanation, but to date, available data do not make it possible to conclude if they act independently from urbanicity, or as part of the effect of urbanicity on psychotic disorders. Finally, several studies have consistently shown significant associations between the polygenic risk score for schizophrenia and urbanicity, with several possible explanations (pleiotropic effects, results of prodromic symptoms, or selection/intergenerational hypothesis). Thus, more studies are needed to understand the factors that explain the association between urbanicity and the risk of psychotic disorders. Further studies should account for the interdependence and/or interactions of different psychosocial and physical exposures (as well as gene-environment interactions), and explore this association in low- and middle-income countries.

## Introduction

Non-affective psychotic disorders, i.e., psychotic disorders not mood-related, that include schizophrenia, schizophreniform, schizoaffective, brief psychotic and delusional disorders, are among the most severe and debilitating chronic diseases (Lieberman and First 2018). In 2016, they were estimated to cause 13.4 million years of life lived with disability by the global burden of disease study (Charlson et al. 2018). It is well-known that the incidence and prevalence of psychotic disorders vary widely geographically, on several levels, i.e., between countries/latitudes, between the regions within countries, between the municipalities of the same region, and even between neighbourhoods across cities (March et al. 2008; Jongsma et al. 2019). Several factors have been associated with these geographical variations at different scales (e.g., area deprivation, ethnic density, rate of unemployment, rate of crime, *etc.*) (Richardson et al. 2018; Eaton et al. 2019; Tibber et al. 2019).

Exposure to urbanicity, i.e., the characteristics that define a geographic area as a city (population density, infrastructure, concentration of technology and services, *etc.*), is one of these factors. The population of cities has been on the rise since decades and is estimated to represent 55% of the world’s population in 2018 (and is projected to represent 68% in 2050) (UN 2018). Living in a city offers several benefits (e.g., access to healthcare systems, to cultural resources, transportation, proximity to workplaces, proximity to friends and/or family, access to shops, *etc.*), but may be associated with several diseases, including psychiatric disorders (Krabbendam et al. 2021). However, urbanicity is not a risk factor in itself, but rather a marker for one or several associated risk factors (that will be detailed in the following literature review).

One of the first authors to suggest a link between schizophrenia and urbanicity was Faris and Dunham (1939). Their work investigated the relationship between the spatial distribution of schizophrenia and social disorganisation, which is a construct informed by sociological theories relating to characteristics of urban settings, social issues, and mental disorders including schizophrenia. They found that the inner urban zones of Chicago had the most disorganised and unstable communities, which also had the highest rates of schizophrenia. Since then, many studies in other cities in Western Europe have found a positive relationship between the degree of urbanicity and the risk of psychotic disorders, as well as explored potential factors that may explain this association.

In the present qualitative literature review, we aim to summarise the knowledge about the relationships between non-affective psychotic disorders and urbanicity, focusing particularly on recent findings. First, we summed up the recent epidemiological data regarding links between the level of urbanicity and psychotic disorders – focusing on meta-analyses and recent large studies. We specifically focused on the factors associated with variations of the urbanicity-psychotic disorders association, especially the level of income of the considered countries. We also considered the studies of urbanicity as a modifier factor (i.e., associated with specific characteristics of the disease) of psychotic disorders. Second, we presented factors which could explain the urbanicity-psychotic disorders association, especially psychosocial stressors and physical (air pollution and green space) exposures. Finally, we addressed the studies exploring the putative role of genetic factors in the urbanicity-psychotic disorders association.

### Urbanicity and psychotic disorders: epidemiological state of the art

#### Meta-analyses

Urbanicity has mainly been studied using two different definitions: the population size of the municipality of residence, and population density. Of note, the results of these two methods are highly correlated (March et al. 2008). Vassos et al. (2012) have meta-analysed data from the studies comparing the incidence rates of psychotic disorders according to the level of urbanicity. To avoid inaccuracies in the measurement of the at-risk population (denominator), they restricted their meta-analysis to studies where the study population was national or was based on cohorts that were representative of the general population and which assessed exposure (i.e., the level of urbanicity) at birth or under 15 years of age. Given these stringent selection criteria, only 4 studies were selected. All were conducted in Europe (2 in Sweden, 1 in Denmark, 1 in the Netherlands). The pooled odds ratio (OR) of urbanicity (comparison between the most urban and the most rural environment) from these studies was 2.37 (95% confidence interval (CI) [2.01–2.82]), with a high level of heterogeneity (*I*^2^ = 82%). This increase was statistically significant independently of the definition of the outcome (schizophrenia *vs.* psychotic disorders more broadly), the method of measuring the level of urbanicity, and the time period of exposure considered (birth, upbringing, place of residence at the onset of disease). Interestingly, in the different studies, the level of urbanicity and the risk of psychotic disorders seem to follow a dose-response relationship.

A more recent meta-analysis with broader inclusion criteria (no restriction concerning the study population or the time period of exposure) included 8 studies published between 2005 and 2015 (Castillejos et al. 2018). The authors compared the incidence of psychotic disorders in urban *vs.* rural areas. All studies were conducted in Europe, except one conducted in Taiwan. The mean incidence values were 34.57 (95% CI [10.77-58-36]) per 100,000 persons/year in the urban areas and 16.46 (95% CI [9.15–23.78]) in the rural areas, and the urban/rural incidence rate ratio (IRR) was 2.25, 95% CI [2.00–2.52], which is very similar to Vassos et al. (2012) meta-analysis.

These two meta-analyses have the advantage of synthesising the retrospective cohort studies relative to the influence of urbanicity on the risk of psychotic disorders. However, several interesting and original studies have been published since 2015 (i.e., the end of the inclusion period for Castillejos et al. (2018) meta-analysis). Moreover, these meta-analyses could not provide details on the adjustment factors used in the different studies. Yet these adjustments make it possible to address the key issues regarding individual factors involved in the increase of the risk of psychotic disorders in urban areas.

#### Focus on recent original studies

In the present section, we will focus on 5 original recent studies of the association between urbanicity and the risk of psychotic disorders. These studies were not included in the previously mentioned meta-analyses. They were chosen for their high quality, especially as they studied large samples. They allow for the analysis of methodological details, unlike meta-analyses, particularly concerning the measure of exposure to urbanicity (e.g., the considered age of exposure), as well as the study of statistical adjustment factors. Indeed, these adjustment factors enable reflection on the factors involved in the association between urbanicity and the risk of psychotic disorder.

The first of these recent original studies is a Swedish nationwide longitudinal family-based study of 2.4 million subjects (Sariaslan et al. 2015). The authors examined the impact of to neighbourhood population density and neighbourhood deprivation measured at several points in time (birth, childhood, adolescence) combined with familial risk. Population density at age 15, as well as neighbourhood deprivation, were associated with a higher risk of schizophrenia in the first two regression models (i.e., after adjusting for sex, birth order and birth year), but this association was no longer significant after adjusting for family history of psychosis in first-degree relatives (siblings). Overall, population density and neighbourhood deprivation accounted for 2% of the variance of the risk of schizophrenia. Concerning the explanations of the association between urbanicity and the risk of psychotic disorders, the authors stated that the fact that the associations did not remain significative in the final model (after adjustment on sibling risk) is consistent with a selection hypothesis, where high-risk individuals and families with genetic and environmental liabilities are selected into densely populated or socioeconomically deprived areas (see below concerning the role of genetic factors).

In 2016, Vassos et al. (2016) published a Danish nationwide study on the influence of the level of urbanicity *at birth* on the subsequent risk of several psychiatric disorders, including psychotic disorders. They included all individuals born in Denmark between 1955 and 2006. Urbanicity was measured according to the population size of the municipality of residence. There was a higher risk of psychotic disorders (as well as for most of the psychiatric disorders) in subjects born in the 4 categories of urban areas in comparison to those born in rural areas, with increasing risk according to the level of urbanicity (consistently with a dose-response relationship): IRR = 1.83 for the capital, 1.49 for the capital suburb, 1.14 for provincial cities, and 1.15 for provincial towns.

Interestingly, in another study, Toulopoulou et al. (2017) adjusted for intelligence quotient (IQ), in order to analyse the respective roles of urbanicity and low IQ on the risk of psychotic disorders. They studied a sample of Danish males (*N* = 153,170) from conscription registers, and measured urbanicity between birth and age 10 years according to the population size of municipalities of residence. First, they confirmed the association between the level of urbanicity and the risk of psychotic disorders (IRR = 1.69, 95% CI [1.20–2.38] in the capital city, in comparison to rural areas, but nonsignificant differences for the two other levels of urbanicity). This association was independent of parental age, parental education and occupation, familial psychiatric history, migrant status, and IQ (fully adjusted model: IRR = 1.77, 95% CI [1.25–2.51]) – all of which are important risk factors of psychotic disorders (Wohl and Gorwood 2007; Khandaker et al. 2011; Schürhoff et al. 2020; Selten et al. 2020).

The study of Kirkbride et al. (2017) measured the level of urbanicity at a smaller area level, i.e., at a neighbourhood level. It took place in a diverse – mixed rural and urban – setting, in the East of England (530 administrative neighbourhoods). Outcomes were based on data from early intervention services, and thus concerned subjects 16–35 years old (numerator and denominator). In comparison to the lower level of urbanicity (based on population density), the 3 other levels were associated with significant increases in the incidence of psychotic disorders (IRR = 1.32, 1.36 and 1.71 for the 3 increasing levels of urbanicity, respectively, thus consistent with a dose-response relationship). This result was adjusted for sex, age, ethnicity, socioeconomic status, and neighbourhood-level deprivation. This important study shows that, even at a neighbourhood level, urbanicity is associated with a greater incidence of psychotic disorders.

A recent cohort study of whole people born in Denmark to Danish-born parents between 1972 and 1981 (*N* = 579,039) considered both individual- and neighbourhood-level factors (Pedersen et al. 2022). Individual factors included age, sex, childhood residential transience, and parental variables (Charlson cardiovascular score, death, imprisonment, age, income, education, and employment status), while neighbourhood-level factors included material deprivation, social fragmentation, social marginalisation and physical illness rates. Interestingly, urbanicity was measured (at a neighbourhood level) by both population density and the size of the cities. These measures of urbanicity were both significantly associated with the incidence of psychotic disorders in unadjusted analyses, as well as after adjustment on both individual- and neighbourhood-level factors (median incidence rate and IRR between 1.08 and 1.30, except for population density in the fully adjusted model). This study confirms that both urbanicity measures were associated with psychiatric disorders, and that the studied factors did not fully explain the association.

### The factors associated with variations of the association between urbanicity and the risk of psychotic disorders

#### Methods consideration

The meta-analyses as well as the few studies reviewed above suggest that the positive association between urbanicity and psychotic disorders as well as its effect size are consistent across various methods used to measure the level of urbanicity. Indeed, the association remains significant when urbanicity was measured by population density or by sizes of municipalities of residence. Of note, these two statistical indexes may be highly correlated. Moreover, the effect-sizes of the association did not display important variations according to the method used. Likewise, the period of exposure to the urban environment (birth, childhood, adolescence) did not change the results. These findings are consistent with findings by Pedersen and Mortensen (2001), who demonstrated an absence of effect between various periods of exposure (i.e., the incidence according to the time of exposure was globally stable from birth to age 15). However, it may be that individuals may not move often or that they may move to neighbourhoods that have similar characteristics (e.g., population density), so their exposure to urbanicity may be consistent across age. Therefore, it may be difficult to detect differences of the relationship between urbanicity and psychotic disorders depending on the age of exposure to urbanicity and assess whether there is a specific vulnerability window. Regarding mobility, and specifically mobility to more urbanised areas, several studies show that, when it occurs during childhood, there is an increase in the risk of psychosis (Pedersen and Mortensen 2001; Toulopoulou et al. 2017). However, it is possible the moving in itself is stressful and increases the risk of psychosis in addition to change in urbanicity (Price et al. 2018; Ku et al. 2023). This possible lack of a specific temporal window of sensitivity to the effects of urbanicity is important considering the drift *vs.* causation controversy (Heinz et al. 2013). Indeed, from the time of the initial studies on this topic (Devereux et al. 1940; Hyde and Kingsley 1944), the question of a social drift of subjects with psychotic disorders has been raised. The drift hypothesis states that the association between urbanicity (and other variables, such as deprivation) and psychotic disorders is not (or not only) related to urbanicity causing psychosis, but (or also) to the fact that subjects at risk for psychotic disorders have a tendency to move to more urbanised and/or deprived areas (Hudson 2005; March et al. 2008; Pignon et al. 2019). Thus, according to the drift hypothesis, the association with urbanicity may be a consequence of prodromal or psychotic symptoms, and not a causal phenomenon. A study on relocation of patients with psychotic disorders between 1973 and 2007 in Denmark found a significantly higher rate of rural-to-urban moves (in comparison to rural-urban moves), but the differences were small (16.4% *vs.* 11.4%) (Pedersen 2015). Moreover, as urbanicity measured at birth is predictive of the risk of psychotic disorders, the drift phenomenon could explain only part of this association (Tulloch et al. 2011; Ngamini Ngui et al. 2013).

One important limitation of the scientific literature is that studies are mainly based on healthcare register data. Thus, all studies are only based on treated psychotic disorders. It is known that there is an important treatment gap in the case of psychotic disorders, with a significant rate of subjects suffering from psychotic disorders never receiving care (almost 40% according to Font et al. (2018)). With healthcare services often being more available and/or providing more care in urban settings, this could confound the association between urbanicity and psychosis (and thus be an artefact linked to healthcare service utilisation bias) (March et al. 2008). One strategy which circumvents this limitation is conducting studies in the general population. Regarding psychotic disorders, which are relatively rare (prevalence less than 1% of the population (Saha et al. 2005; Szöke et al. 2015)), such studies might use surrogate measures, i.e., measures that are linked to the outcome and which can be used instead (Szöke et al. 2014). For instance, subclinical psychotic phenomena have been suggested as surrogates, as they are considered to be on the continuum of psychotic disorders (Verdoux and van Os 2002; Linscott and van Os 2013) and occur more frequently. This hypothesis of a continuum of psychosis suggests that at least some of the genetic and environmental risk factors contributing to variations at the highest (i.e., disorder) level of the continuum also have an impact at lower levels. Thus, according to this hypothesis, the level of urbanicity may also impact the rate of subclinical psychotic phenomena in the general population. These outcomes, as they do not meet the criteria for characterised disorders and lead, to a lesser degree, to functional impairment are not (or at least less) impacted by the healthcare utilisation selection bias.

Several studies have investigated the association between urbanicity and subclinical psychosis, starting with van Os et al. (2001), who found associations between urbanicity and delusions and/or hallucinations (OR = 1.47, 95% CI [1.25–1.72]), as well as overall psychotic symptoms (OR = 1.18, 95% CI [1.13–1.24]) in the general population. Solmi et al. (2020) found a significant association between population density and the level of positive psychotic symptoms in 18 years-old subjects (most densely *vs.* least: OR = 1.59, 95% CI [1.15–2.21]) in the Avon Longitudinal Study of Parents and Children (ALSPAC) cohort. In a children’s cohort study, also based in the United-Kingdom (UK), the neighbourhood-level urbanicity was associated with greater psychotic symptoms (age-5: OR = 1.80, 95% [1.16–2.77], age-12: OR = 1.76 95% [1.15–2.69]) (Newbury et al. 2016). Such studies provide an argument against the idea of a healthcare service utilisation bias.

#### Low and middle-income countries

One other major limitation of the scientific literature is that studies were exclusively conducted in Western Europe (with the exception of one study conducted in Taiwan (Chien et al. 2004)). Of note, no incidence studies concerning the relationships between psychotic disorders and urbanicity has ever been carried out in the United States of America (USA), only studies that examined subclinical psychosis and found inconsistent results (Shevlin et al. 2011; Oh, Koyanagi, et al. 2020; Oh, Susser, et al. 2020; DeVylder et al. 2023). To our knowledge, no studies on the influence of urbanicity on the incidence of psychotic disorders have been conducted in low- and middle-income countries. Several studies have aimed to explore the association between urbanicity and the *prevalence* of psychotic disorders, particularly in China (*cf.* details below) (Chan et al. 2015). Prevalence studies should be interpreted with caution, as several phenomena make prevalence less precise than incidence for assessing risk and, consequently, for studying the link between the disease and an area-level factor like urbanicity, even for chronic diseases as psychotic disorders: higher mortality among people with the disease, remission or migration of people with the disease from the catchment area, or the absence stability of the population (Grimes and Schulz 2002). Three of these phenomena (mortality, remission, migration) can bias the rate downward, and prevalence studies should consider them when studying aetiology issues, e.g., using incidence-prevalence models (Saha et al. 2008; Pignon, Schürhoff, Baudin, et al. 2018). On the other hand, specifically, regarding urbanicity, the phenomenon of moving to cities could occur after the onset of the disease (Pedersen 2015). However, prevalence studies are useful to assess the severity of a disease and/or the comorbidities according to clinical or biological factors and can provide important insights on factors associated with different courses of the disease, i.e., modifiers of a disease (Stolk et al. 2008). DeVylder et al. (2018) have studied a World Health Organisation (WHO) cross-sectional survey, that included nationally representative samples of adults residing in 42 low and middle-income countries to measure the association between psychotic symptoms and urbanicity. Urbanicity was measured at the time of the survey and was defined using a dichotomous variable. Among the 215,682 subjects, urbanicity was not associated with the prevalence of psychotic disorder (OR = 0.89, 95% CI [0.76–1.06]), nor of psychotic symptoms (OR = 0.99, 95% CI [0.82–1.15]). Another study on the prevalence of psychotic disorders which took place in Mozambique, found decreased rates in urban settings, in comparison to rural areas (Patel et al. 2007).

Moreover, in addition to the cross-national study of DeVylder et al. (2018), regarding low- and middle-income countries, several articles studying subclinical psychosis have been published, starting with a study conducted in South Africa (Temmingh et al. 2011). This study measured the association between the prevalence of hallucinations and urbanicity (dichotomized between urban and rural areas) in a sample of the general population. The prevalence of hallucinations (i.e., positive psychotic symptoms) was not significantly different according to the urbanicity of the place of residence (13.1% in urban areas, *vs.* 12.4% in rural areas). In Turkey, Binbay et al. (2012) analysed a sample representative of the general population, and found results consistent with a dose-response relationship between urbanicity and the severity of psychotic phenomena (subclinical psychotic experiences: OR = 1.14, 95% CI [0.95–1.37], low-impact psychotic symptoms: OR = 1.41, 95% CI [1.04–1.91], high-impact psychotic symptoms: OR = 1.59, 95% CI [1.59–1.15–2.21], clinical psychotic disorder: OR = 1.65, 95% [1.09–2.53]).

Two studies with subclinical outcomes have recently been published among participants in China, which has been experiencing rapid urbanisation recently. In a large (*N* = 4132) representative survey of young adult men in the Sichuan Province, Coid et al. (2018) investigated the effects of urbanicity, work migrancy, and residential stability on the prevalence and severity of psychotic symptoms. They found an overall high prevalence of psychotic symptoms (31.1%). In univariate analyses of this cross-sectional study, both urban birth and urban living were associated with reporting 3 or more psychotic symptoms (aOR = 1.90, 95% CI [1.28–2.82] for urban birth, aOR = 2.05, 95% CI [1.46–2.88] for urban living). Adjusted analyses found that, in comparison to subjects without psychotic symptoms, men experiencing 5 psychotic symptoms or more had higher rate of urban birth (aOR = 8.51, 95% CI [1.49–49.48]). Other exposures (urban *living*, migrant status, residential stability) were not associated with psychotic symptoms. Another study of undergraduate students in the Chengdu (Sichuan Province again) found opposing results, with students who grew up in rural settings displaying higher rates of psychotic symptoms (OR = 1.41, 95% CI [1.22–1.63]) (Wang et al. 2019). Chan et al. (2015) analysed the longitudinal variations in the prevalence of psychotic disorders between urban and rural areas between 1990 and 2010 in China, i.e., during a period of urbanisation and industrialisation, using data from 42 prevalence studies. This analysis showed that, in 1990, the lifetime prevalence rates were similar between urban and rural areas (0.39% in urban China, 0.37% in rural areas), while in 2010 the prevalence increased more in urban settings (0.83% *vs.* 0.50%).

To explain the absence of association between urbanicity and psychotic outcomes in low- and middle-income countries, and the discrepancies with high-income countries, DeVylder et al. (2018) discuss the role of several factors. They hypothesised that economic deprivation and social isolation may be more frequent in the cities of high-income countries, or that may be greater rural-urban differences; additionally, protective factors as familial and social cohesion may be stronger in the cities of low- and middle-income countries. Another factor is the proportion of migrants or subjects from ethnic minorities, which is higher in urban areas of high-income countries, as well as the ethnic minority effect (for more details concerning migrant and ethnic effects, see below). However, many low- and middle-income countries also experience migration and refugee phenomena – refugees being particularly at risk for psychotic disorders (Hollander et al. 2016). Another hypothesis is the use of cannabis or other psychoactive substances, which seems to be more frequent in cities in high-income countries (Coughlin et al. 2019).

### Urbanicity: a modifier factor for psychotic disorders?

Modifier factors as opposed to risk factors, are not characterised by an association with an increased level of risk, but with specific characteristics of the disease, such as clinical severity, course or prognosis. Few studies have considered urbanicity as a modifier factor, and their results are inconsistent.

Analysing the European Network of National Schizophrenia Networks Studying Gene-Environment Interactions (EU-GEI) study data, Quattrone et al. (2019) have shown that among subjects with a first episode of psychosis (FEP), there is a positive association between urbanicity (as measured by population density) and general psychopathology (*ß* = 0.30 95% CI [0.24–0.36]) as well as symptoms from the negative (*ß* = 0.12 95% CI [0.05–0.19]) dimensions of the PANSS (positive and negative syndrome scale), without significant associations with symptoms from the other dimensions (i.e., positive, disorganisation, manic, depressive). In another FEP sample from the UK, neighbourhood-level urbanicity (measured by population density) was not associated with any clinical dimension of psychosis (Tibber et al. 2019).

Finally, in a large Danish population-based cohort of all FEP subjects between 1996 and 2016, Wimberley et al. (2016) found that urbanicity was associated with lower rates of 5-year treatment resistance (i.e., the rate of clozapine prescription). Indeed, in comparison to subjects from areas with a higher level of urbanicity, patients from provincial and rural areas displayed higher rates of resistance in FEP subjects (hazard ratios (HR)=1.44 95% CI [1.31–1.59] and HR = 1.60 95% CI [1.43–1.79], respectively). Several explanations of this result have been proposed such as the higher density of healthcare structures in cities, which could reduce the duration of untreated psychosis (DUP) and in turn the level of resistance, or differences in prescribing practices across different levels of urbanicity. However, this may also be an indication of different clinical subtypes in rural versus urban areas.

Overall, based on these studies, urbanicity could not be considered as associated with a more severe clinical course of psychotic disorders and, on the contrary, may be associated with lower severity.

### Which underlying factors can explain the association between urbanicity and the risk of psychotic disorders?

In the first section of this article, we reviewed the main epidemiological studies looking at the association between urbanicity and risk for psychotic disorders. However, this association is not considered as directly causal, with urbanicity being a marker for one or several other risk factors. We will now review the different hypotheses about specific factors characterising urban settings which may explain the association with psychotic disorders. First, we will review psychosocial stressors, especially neighbourhood-level factors, as well as migrant and ethnic factors. Second, physical exposures will be reviewed, and specifically the role of air pollution and exposure to green space.

#### Psychosocial stressors

The associations between psychosocial stress, such as childhood trauma, discrimination experiences or stressful life events and psychotic disorders is well known, with several psychosocial stressors being consistently associated with an increased risk for psychotic disorders in longitudinal studies (Varese et al. 2012; Beards et al. 2013; Pearce et al. 2019). Several of these stressors may be present at an increased level in cities in comparison to rural areas, and thus could account for the effect of urbanicity on the risk of psychotic disorders.

#### Social fragmentation and social capital: neighborhood-level studies

Social stress has been mentioned from an early stage to explain higher rates of incidence of psychotic disorders in cities (Faris and Dunham 1939; Heinz et al. 2013). Social stress in cities may be measured at the neighbourhood level. Social fragmentation is considered to be one of the best markers of social community related stress. It is defined as the disrupted connections between individuals and society, and have been measured with various area-level characteristics, including the percentage of private renters, single people, single parent households, those who are divorced, and those who have changed their address in the previous year (residential instability) (Allardyce et al. 2005; Pignon et al. 2016; Szöke et al. 2016). These phenomena are more present in cities in comparison to rural areas (Allardyce et al. 2005; Kirkbride et al. 2008). Five studies reported associations between social fragmentation and the incidence of psychotic disorders, and all but one reported significant associations, with a wide range of OR (from 1.42 to 12.84) (Ku et al. 2021). Interestingly, analysing whole Scotland data, Allardyce et al. (2005) reported a dose-response relationship between social fragmentation and the rate of first admissions for psychotic disorders. Similarly, exposure to area-level residential instability (that is related to social fragmentation) during childhood and adolescence has been shown to predict the onset of psychosis even among those who never moved themselves (Ku et al. 2020, 2022). Moreover, two five studies have concomitantly studied the level of urbanicity (Allardyce et al. 2005; O’Donoghue et al. 2016); and when adjusting for social fragmentation, urbanicity was no longer significantly associated with the risk of psychotic disorders.

Social capital has been considered relevant in the aetiology of psychotic disorders and is measured by the level of civic participation, social networks or trust, and to give shape to the quality and quantity of social interaction (McKenzie et al. 2002; Sartorius 2003). Kirkbride et al. (2008), in the Aetiology and Ethnicity in Schizophrenia and Other Psychoses (ÆSOP) study, which took place in 33 neighbourhoods of South London, reported a non-linear association between the level of social capital and the incidence of psychosis: they found that both low (IRR = 2.0, 95% CI [1.2–3.3]) and high (IRR = 2.5, 95% CI [1.3–4.8) levels of social capital were associated with higher incidence of psychotic disorders. Neighbourhoods with low social capital may fail to mediate social stress, whereas high social capital neighbourhoods may increase the risk of psychosis for residents who are excluded from accessing available social capital. Remarkably, the level of social capital was negatively correlated with population density. Moreover, as for social fragmentation, after adjustment on social capital, the association between urbanicity (measured as population density) and psychotic disorders was not significant. In another cohort study of 4.5 million people in Sweden, social capital (as assessed by the rate of voting participation) was associated with the risk of first hospital admission for psychotic disorders (low level: OR = 2.89 95% CI [2.72–3.07] among men, OR = 2.62 95% CI [2.47–2.78]; unfortunately, they did not adjust for urbanicity) (Lofors and Sundquist 2007). Of note, in control subjects from the EU-GEI study, low social capital was associated with higher levels of subclinical psychosis (but social capital was measured at an individual, not neighbourhood level) (Pignon et al. 2021, 2022).

Overall, the social environment, as measured by neighbourhood-level characteristics including social fragmentation and social capital, may partly explain the association between urbanicity and psychotic disorders. Other characteristics of the social environment such as social inequality and social deprivation have been shown to be associated with a greater incidence of psychotic disorders (Kirkbride et al. 2014; Kirkbride, Hameed, Ankireddypalli, et al. 2017). However, to our knowledge, it is not known whether these other environmental characteristics may explain the urbanicity effect on psychosis.

#### Migration and ethnic minorities

Many studies have shown that first- and second-generation migrants (and more recently, third-generation), as well as people belonging to ethnic minority groups, are at higher risk of psychosis (Cantor-Graae et al. 2005; Bourque et al. 2011; Amad et al. 2013; Pignon, Schürhoff, Szöke, et al. 2018; Tortelli et al. 2018; Selten et al. 2020). Migrants and those belonging to ethnic minorities often live in urban areas and in poor living conditions, with high residential mobility (Eurostat 2015; Fett et al. 2019). They may be exposed to experiences of discrimination, which have been shown to be associated with an increased risk of psychotic disorders and a higher level of subclinical psychosis (Bardol et al. 2020; Pignon et al. 2021). However, several studies have shown that a higher ethnic density effect (i.e., the neighbourhood proportion of people from the same ethnicity) has a protective effect on the level of risk of psychotic disorders among ethnic minorities (Veling et al. 2008; Bécares et al. 2009; Shaw et al. 2012). This protective effect is hypothesised to be linked to social support and to reduced acculturation stress.

Interestingly, in the EU-GEI multinational incidence study, in a multivariable model including both ethnicity and urbanicity, ethnic minority status was associated with greater incidence of psychotic disorders (IRR = 1.59, 95% CI [1.46–1.72]), while urbanicity was not (IRR = 1.01, 95% CI [0.99–1.02]) (Jongsma et al. 2018). Of note, the EUGEI study included countries from Southern Europe (Italy and Spain) and Brazil. However, a longitudinal study in all of Denmark over the course of 38 years simultaneously examined the effects of migrant/ethnic status, urbanicity, neighbourhood exposures, and parental background (i.e., history of psychiatric disorders and socio-economic level) in childhood (Schofield et al. 2017). All of these factors were found to be significantly associated with incidence, including neighbourhood urbanicity (IRR = 1.13, 95% CI [1.11–1.14]). Several other studies have also considered both ethnic/migrant status and urbanicity in the same models, and found independent effects of these variables (Kirkbride, Hameed, Ioannidis, et al. 2017; Richardson et al. 2018), suggesting that other factors may explain the urbanicity-psychosis association.

These studies confirm the role of neighbourhood- and individual-level factors, including social stressors. However, the current state of our knowledge is not sufficient to determine whether there are independent effects of migrant/ethnic factors and urbanicity or if migrant/ethnic factors account for a part of the urbanicity effect (Heinz et al. 2013).

### Experimental data

One virtual reality study aimed to model the effect of urbanicity and environmental social stress on level of distress, and its subsequent link with psychosis (Veling et al. 2016). The authors included subjects with different levels of risk for psychosis (subjects with a psychotic disorder, subjects at ultra-high risk for psychosis (UHR), siblings of subjects with psychotic disorders, and controls), and exposed them to different levels of 3 different putative stressors: population density (number of avatars in the scene), ethnic density, and hostility (*via* the facial expression of avatars). They measured the level of social distress, as well as the presence of paranoid thoughts. Two main findings are interesting with regard to urbanicity. First, of the 3 putative stressors, only ‘population density’ was associated with both distress and paranoia. This result is consistent with a direct effect of urbanicity on the level of stress. Second, the level of risk for psychosis had an influence on the reaction to the stressor: high psychosis risk, as well as pre-existing affective and/or psychotic symptoms, were associated with more paranoia and distress in social environments. This result shows that urbanicity interacts with other factors, such as the pre-existing level of vulnerability for psychosis. This vulnerability probably involves deficits in social cognition, which may have more severe effects in densely populated urban areas characterised by a high frequency of social encounters (Green et al. 2015; Krabbendam et al. 2021).

### Air pollution and the use of green space

#### Air pollution

The role of air pollution is now well-established as a risk factor for several non-psychiatric diseases, such as cardiovascular and pulmonary diseases, or cancer (WHO 2018). Exposure to several pollutants, such as particulate matter (PM) with a diameter of less than 2.5 (PM_2.5_) 10 micrometres (PM_10_), nitrogen oxide (NO_X_) and nitrogen dioxide (NO_2_), is assumed to be among the main causal factors of this association (Stieb et al. 2021; Mohammadi et al. 2022; Münzel et al. 2023). Aside from the aforementioned diseases, air pollution has also been linked to neurological diseases to have neurotoxic effects in several animal studies (Leiva et al. 2013; Buoli et al. 2018). As it is partly related to traffic and heating systems, air pollution is known to be higher in urban settings, suggesting potential involvement in the association of urbanicity and psychotic disorders. Surprisingly, the impact of pollution has been less studied than other (e.g., stress) factors. Several studies have shown that air pollution peaks (short-term exposures, especially PM peaks) were associated with a higher number of emergency visits for psychotic disorders (Gao et al. 2017; Bernardini et al. 2020; Pignon et al. 2022), but there are fewer studies of the effects of long-term exposure to air pollution. We will review these longitudinal studies next.

The first study of the influence of long-term air pollution exposure was published in 2004, and was based on the level of air pollution at birth of 7455 children born in Denmark (Pedersen et al. 2004). The findings showed a significant association between schizophrenia and the level of benzene (RR = 3.20, 95% CI [1.01–10.12]). This association was no longer significant after adjustment for the degree of urbanisation. The second study concerning long-term exposure to air pollution and psychotic disorders was published only in 2019 (Khan et al. 2019). This study analysed data from the USA and Denmark. The Danish study was based on a health outcome registry, and analyzes data pertaining to the influence of *childhood-exposure* (first 10 years of life) to global air pollution (based on 14 air quality indicators) in more than 1,400,000 subjects. They found a significant impact of air pollution: comparing the groups having the highest *vs.* lowest childhood exposure to air pollution, the incidence of psychotic disorders was increased (+148%, 95% CI [119%–180%]). Adjusted models showed that both air pollution and urbanicity remained associated with the incidence of psychotic disorders. The US data were different, as air pollution was estimated at a county-level (thus not individual), and exposure to air pollution was estimated based on the years 2000 to 2005, whereas prevalence was measured between 2002 and 2013 (Ioannidis 2019; Pignon et al. 2020). After adjustment for (among others) population density, the findings did not show any significant differences regarding the prevalence of psychotic disorders. Taken together, the two studies suggest that the window of vulnerability is in childhood.

Two other Danish longitudinal studies of the impact of childhood exposure to air pollution have been published more recently. Antonsen et al. (2020) followed all subjects born between 1980 and 1984 in Denmark, and revealed a significant association between mean daily exposure to NO_2_ and NO_x_ at subjects’ residential addresses from birth to their tenth birthday with the risk of psychotic disorders: IRR per 10-μg/m^3^ increase of NO_2_ = 1.20 (95% CI [1.09–1.33]), and for NO_x_ = 1.07 (95% CI [1.02–1.10]). Interestingly, these results were adjusted for the level of urbanicity; and the degree of urbanisation, which was associated with schizophrenia, was no more associated after adjustment for NO_2_. In the second study, the authors studied both childhood exposure to NO_2_ and the polygenic risk of schizophrenia (PRS-SZ) in a random sample of 30,000 subjects (Horsdal et al. 2019). Consistent with the Antonsen et al. (2020) study, a 10-μg/m^3^ increase in childhood daily exposure to NO_2_ was associated with an elevated risk of psychotic disorders (adjusted HR = 1.27, 95% CI [1.19–1.35]). Moreover, although childhood exposure to NO_2_ was associated to the PRS-SZ, this exposure remained associated with the incidence of psychotic disorders after adjustment on PRS-SZ (see below for more information on the role of genetic factors).

The last longitudinal study used subclinical outcomes in a population-based cohort of 2232 children followed until 18 years of age in England and Wales, and found significant associations with exposures to NO_2_ (comparison between the lowest and the highest quartile: OR = 1.71, 95% CI [1.28–2.28]), NO_X_ (OR = 1.72, 95% CI [1.30–2.29]), and PM_2.5_ (OR = 1.71, 95% CI [1.11–1.90]) (Newbury et al. 2019). Interestingly, the authors show that exposures to NO_2_ and NO_X_ explained 60% of the association between urbanicity and adolescent psychotic symptoms. Overall, these findings show that air pollution may partly explain the urbanicity-psychotic disorders association.

#### Green space

Green space (parks, grasslands, forests, *etc.*) have recently been studied with regard to mental health, and particularly where psychotic disorders are concerned. The proximity to green space may influence the risk of psychotic disorders by promoting social connection and physical exercise, and/or by decreasing stress, noise and the level of exposure to air pollution. Green space is less common in urban areas (Nieuwenhuijsen et al. 2022). Moreover, it is known that subjects with psychotic disorders have a significantly lower amount of green space in their neighbourhood (Boers et al. 2018). Of note, the level of green space in a given neighbourhood may also be associated with several important variables, such as socio-economic level, or amount of physical exercise.

Two recent Danish nationwide population-based studies have aimed to determine the influence of childhood exposure to green space (as modelled by the normalised difference vegetation index (NDVI), i.e., the amount of green space around a residence) on the risk of psychotic disorders. The first followed 943,027 subjects born in Denmark between 1985 and 2003 until 2013 (Engemann et al. 2018). Children living in the lowest decile of green space (at age 10) had a higher risk for psychotic disorders (IRR = 1.52, 95% CI [1.36–1.69]). The results were consistent with a dose-response relationship, and the effect was stronger when the NDVI was considered during early childhood, and even stronger when it was considered cumulatively between age 0 and age 10. Remarkably, after adjustment on urbanicity, the effect of green space was lower but remained significant (IRR = 1.29, 95% CI [1.20–1.40]).

The second study aimed to examine the association between the risk for psychotic disorders and both the level of childhood exposure to green space and the PRS-SZ, regarding gene x environment (GxE) interaction (Engemann et al. 2020). The authors studied a random sample of 19,746 subjects born between 1981 and 2005, and followed until 2012, and also considered the 10 first years of life. Lower NDVI and higher PRS-SZ were both associated with the risk of psychotic disorders (*p*-values < .001), with dose-response relationships. Moreover, they were slightly but significantly inversely correlated (*ρ* = −0.0779, *p*-values < .001). After adjustment on PRS-SZ, an increase of NDVI values remained associated with a decreased risk for psychotic disorders (adjusted HR = 0.52, 95% CI [0.40–0.66]). Adjustment for family history of psychiatric disorders, parent’s socioeconomic status, place of residency, and green space, did not change the significance of the association (there was only a slight attenuation). However, together, PRS-SZ and NDVI explained only 2.40% of the variance of the outcome (no GxE interaction, but additivity of PRS-SZ and NDVI effects).

These significant associations – and especially the dose-response relationships – are arguments in favour of a protective effect of green space use during childhood, and provide interesting insight with regard to the effect of urbanicity on the risk of psychotic disorders.

Noise pollution (higher in urban settings) has to our knowledge not yet been studied with regard to psychotic disorders. However, we can hypothesise that it could induce some stress and have, contrary to the use of green space, a risk factor effect on psychotic disorders. Consistently with this hypothesis, noise pollution has already been negatively associated with cognitive functioning and reduced wellbeing (Krabbendam et al. 2021).

### Other environmental factors

#### Cannabis use

Among other factors suggested as possible explanations for the effect of urbanicity on the risk of psychotic disorders, cannabis is often mentioned (Heinz et al. 2013; Paksarian et al. 2018). It is one of the first environmental factors to be identified as being associated with psychotic disorders (Andreasson et al. 1987). The issue at hand is not the relationship between cannabis use and the risk of psychotic disorders, which have been consistently associated in several studies (Moore et al. 2007; Marconi et al. 2016; Di Forti et al. 2019), but rather the higher use of cannabis in urban settings as compared to rural areas. To date, this issue has been poorly studied, except in the USA. For instance, Coughlin et al. (2019) showed differences between urban and rural rates of monthly cannabis use during the years 2007–17 (9.75% *vs.* 8.19%: aOR = 0.71 95% CI [0.68–0.75] with urban areas as a reference). In a survey of 171,766 American adults (2015–18), the rate of past-year cannabis use was also lower in small or non-metropolitan areas (in comparison to large metropolitan areas: OR = 0.92 95% CI [0.87–0.97] for small metropolitan, and OR = 0.77 95% CI [0.71–0.83] for nonmetropolitan areas) (Moore et al. 2021). Other American data, especially from the 3 phases of the National Epidemiologic Survey on Alcohol and Related Conditions (NESARC, surveys in the general population), were consistent with these magnitudes of difference of prevalence in the recent use of cannabis (Hasin et al. 2019). Overall, these differences seem too slight to completely explain the effect of urbanicity on the risk of psychotic disorders. Moreover, the association between urbanicity and cannabis use could be dependent on the countries and areas of study. Interactions between these two factors (cannabis and urbanicity) may be involved. Studying subclinical phenomena (psychotic symptoms) in a German longitudinal study of young (14–24 years-old) subjects (*N* = 1923), Kuepper et al. (2011) found that the effect of cannabis use on incident psychotic symptoms was stronger in subjects who grew up in urban settings (consistent with a Environment x Environment interaction phenomenon).

#### Infectious diseases

Severe childhood infectious diseases, both bacterial and viral, are known to be risk factors for psychotic disorders, and several longitudinal studies have found consistent significant associations (Benros et al. 2011; Khandaker et al. 2012; Nielsen et al. 2014). As social contacts are higher in cities, infections should theoretically be more frequent. Dalman et al. (2008), in a Swedish national cohort of 1.2 million children born between 1973 and 1985, found associations between viral infections of the central nervous system (CNS) and (i) urbanicity (RR = 1.6, 95% CI [1.4–1.8]), and (ii) risk of psychotic disorders (RR = 1.5, 95% CI [1.0–2.4]). Adjusted for urbanicity, the risk associated with CNS infections was no longer significant. These results are consistent with a possible involvement of childhood infections in the urbanicity-psychotic disorders association.

### The role of genetic factors

Several studies have examined the relationships between the effects of genetic factors in the association between urbanicity and the risk of psychotic disorders. GxE interaction can be defined as a different effect of an environmental exposure on disease risk in persons with different genotypes. Van Os et al. (2003, 2004) studied the role of familial history of psychotic disorders, which could be considered as a proxy of genetic vulnerability for psychotic disorders. They found synergistic (i.e., additive, or independent) effects of urbanicity and familial history of psychotic disorders: the effect of urbanicity was stronger among subjects with a familial history of psychotic disorders, or the effect of a familial history of a psychotic disorder was stronger in more urban settings. To our knowledge, no studies found evidence for GxE interaction with urbanicity considering multiplicative interaction. A multiplicative GxE interaction involves, beyond the additive/independent effects of each genetic and environmental factors, a specific effect of the combination of both genetic and environmental factors (Rothman et al. 1980; VanderWeele and Knol 2014).

Several studies also analysed direct genetic-environment association, i.e., the relationships between urbanicity and genetic risk factors for psychotic disorders (independent of the risk for psychotic disorders itself). Several such studies found gene-environment associations for other environmental factors, e.g., cannabis or childhood trauma, which were associated with PRS-SZ (Gage et al. 2017; Woolway et al. 2022). These associations disrupt the nature/nurture dichotomy, as they raise the issue of environmental factor exposure being conditioned by genetic factors (Pingault et al. 2018). Using several samples, including the UK Biobank, Dutch and Australian data (*N* > 500 k subjects), PRS-SZ was significantly associated with the population density of the subjects’ current place of residence (*r*^2^ = 0.12%, *p*-value** ***=*** **5.69 × 10^−5^) (Colodro-Conde et al. 2018). Another study in Denmark found similar results (Paksarian et al. 2018). In another study of the UK Biobank cohort (*N* = 207,963), Maxwell et al. (2021) found that the PRS-SZ was associated with higher birthplace population density (*p-value*** **=** **8.10^−5^).

Two other studies have analysed the relationships between PRS-SZ, urbanicity and several factors considered to be involved in the urbanicity-psychotic disorders association. In a cohort of 2232 British twins followed to age 18, PRS-SZ was associated with urbanicity at age 18, as well as familial poverty, residential mobility and neighbourhood variables (rates of crime and neighbourhood disorder) (Newbury et al. 2022). The second study (already presented above) found a correlation between the childhood NO_2_ exposure and the PRS-SZ (*p-*value** **<** **.001) (Horsdal et al. 2019). Moreover, adjustment for PRS-SZ attenuated the effect of childhood NO_2_ exposure, but did not make it disappear. These two studies are consistent with associations between PRS-SZ and the level of urbanicity.

Several hypotheses can be proposed to explain these associations, summarised by Maxwell et al. (2021). They may be driven by pleiotropic effects of genetic factors, which could affect both the risk for psychotic disorders and the choice of residence. Genetic factors could also indirectly influence choice of residence through an influence on personality traits or behaviours, such as creativity or risk taking. Another explanation is similar to the inverse causality hypothesis of the cannabis-psychotic disorder association: prodromic symptoms of psychotic disorders, as positive delusional symptoms or social withdrawal, that may be associated with PRS-SZ may motivate subjects who will go on to develop schizophrenia to move towards urban areas (Smigielski et al. 2021). The selection hypothesis and intergenerational processes could also be involved, especially social drift: subjects with psychotic disorders (or subclinical psychotic phenotype) may move to urban areas, explaining the association between the PRS-SZ of their offspring and the level of urbanicity of their residence at birth or during childhood. More data are necessary to explore these different hypotheses. Moreover, the major limitation of PRS-SZ is the lack of generalisability, since it concerns only Caucasian subjects, which limits the conclusions concerning the study of the role of social environment factors.

## Discussion

Urbanicity is a very important factor to study with regard to psychotic disorders, particularly given the phenomenon of rapid urbanisation, and thus a potentially important attributable aetiological fraction. Moreover, the study of urbanicity and of the various factors explaining an association with psychotic disorders has heuristic value with regard to their aetiology.

Longitudinal studies of the impact of exposure to urbanicity on the risk of psychotic disorders have consistently shown a significantly positive association, with a relative risk between 2 and 2.5 in the meta-analyses (and lower rates according to recent original studies). This association has been significant regardless of the time of exposure of urbanicity (birth, childhood, adolescence) or the definition of urbanicity (based on population density or on the size of the municipalities). Most of the original studies were conducted in Western Europe, and no incidence studies have yet been conducted in low- and middle-income countries. In addition, to date, urbanicity cannot be considered as a specific determinant of course/severity/prognosis of illness among subjects with psychotic disorders, as studies have found inconsistent results.

With regard to the factors explaining the significant association in European studies, neighbourhood-level social fragmentation and social capital may be determinants, as opposed to individual-level migrant or ethnic status. Physical exposures to air pollution (positive association) and green space (negative association) may also be part of the explanation, but the available studies do not allow for us to conclude if these factors act independently from urbanicity, or if they partly explain the effect of urbanicity on psychotic disorders. Moreover, childhood infectious diseases could also account for the effect of urbanicity.

Finally, several studies have consistently shown statistically significant associations between PRS-SZ and urbanicity, with several possible explanations (pleiotropic effects of genetic factors, results of prodromic psychotic symptoms, or a selection/intergenerational hypothesis).

Further studies on this topic have several challenges to address, starting with the lack of incidence studies in low- and middle-income countries. In addition, the role of the underlying factors explaining the urbanicity-psychotic disorders association needs to be specified. Studies should account for the interdependence of exposures (e.g., psychosocial and physical exposures). Moreover, the role of genetic factors and of putative gene-environment interactions should be investigated. The role of air pollution is not clear, especially the involved air pollutants, and which time-vulnerability windows are pertinent. Currently, only outdoor pollution has been studied, with approximate measures of exposure. Indoor pollution, studied for other diseases (e.g., cancer (White et al. 2018)), has never been explored with regard to psychiatric disorders.
